# Predictors of Revictimization in Online Dating

**DOI:** 10.1177/08862605211073715

**Published:** 2022-02-28

**Authors:** Fatemeh Fereidooni, Judith Daniels, Miriam Lommen

**Affiliations:** 1 Department of Clinical Psychology, 3647 University of Groningen, Netherlands

**Keywords:** childhood maltreatment, sexual victimization in adulthood, revictimization, online dating, affect/self-esteem regulatory sex motives

## Abstract

**Introduction:** While a significant association between childhood maltreatment and sexual victimization in adulthood has been established in previous research, it is unknown whether this also applies to the context of online dating. Therefore, we aimed to investigate whether revictimization is common in online users and which mechanisms mediate this risk. **Method:** The participants were 413 heterosexual women aged between 18 and 35 who used mobile dating applications in the year before the assessment. The participants reported information on using mobile dating applications, motives for engaging in casual sex, protective dating strategies, and general motives for online dating. **Results:** Childhood maltreatment severity was positively related to both cyber and in-person sexual victimization severity. Motives related to regulating negative affect and self-esteem mediated the relationship between childhood maltreatment severity and in-person sexual victimization severity in adulthood. Furthermore, those motives moderated the association between cyber and in-person sexual victimization. The effect of cyber victimization on in-person sexual victimization was stronger at higher levels of affect/self-esteem regulatory sex motives compared to lower levels. The affect/self-esteem regulatory sex motives were not related to protective dating strategies. **Discussion:** The results of the study imply that a history of childhood maltreatment is a risk factor for sexual victimization in adulthood among young heterosexual women who use online dating. One of the factors linking these variables in this population might be affect/self-esteem regulatory sex motives. Future studies should aim at replicating these associations prospectively.

## Introduction

Childhood maltreatment is associated with a higher risk of revictimization in adulthood (Werner et al., 2016). In a large Dutch sample, 50% of women and 30% of men with a history of childhood sexual abuse reported sexual revictimization in adulthood (de Haas et al., 2012). A meta-analytic review also showed a positive relationship between childhood maltreatment and intimate partner violence victimization (Li et al., 2019). While the association between childhood maltreatment, including sexual abuse, and (sexual) victimization in adulthood is well established, it remains unclear whether it also translates to online dating. Childhood maltreatment might be associated with both cyber victimization (victimization via the internet or electronic technologies) and in-person victimization among online dating users. Addressing this question is important for three reasons. First, online dating is widely used. In a study, approx. half of the participants between the ages of 18–29, recruited via advertisements on Facebook, were currently using the online dating application *Tinder* (Timmermans & Courtois, 2018). Second, prior studies support high risk of sexual victimization in online dating. The risk of sexual victimization seems to be 2–3 times higher in online dating users compared to non-users in student samples (Choi et al., 2018a; Shapiro et al., 2017). In addition, a study on people contacting sexual assault centers in the Netherlands between 2013 and 2020 reported that seven percent of the victims met the perpetrator via the internet (Hiddink-Til et al., 2021). Third, online dating users seem to show risky sex behavior more frequently than non-users. For instance, they report having a higher number of sexual partners (Choi et al., 2016b), engaging more often in casual sex, exhibiting vague communication of sexual intentions, and using alcohol in sexual situations (Tomaszewska & Schuster, 2020). This risky sex behavior might explain the increased risk of sexual victimization among the users.

Importantly, prior studies show a relationship between childhood sexual abuse and risky sex behavior (Abajobir et al., 2017). One of the theories trying to explain this association is traumatic sexualization theory (Finkelhor, 1988). In this theory, it is presumed that people who have been victimized sexually in childhood use sex for meeting their non-sexual needs such as receiving other’s attention. Similar to this formulation, Orcutt et al. (2005) theorize that people with a history of childhood sexual abuse use sex as an emotion regulation strategy to reduce negative affect. This formulation differs from the deficit-focused conceptualization of revictimization (Messman-Moore & Long, 2003), which suggests that PTSD symptoms such as numbing or hyperarousal might interfere with risk detection and risk reaction, which in turn might result in revictimization. Instead, Orcutt et al. (2005) assume that the strong urge to reduce negative affect by engaging in risky sex behavior is consciously given priority in potentially risky situations, for example resulting in a higher probability of sex with strangers (Miron & Orcutt, 2014). Another motive for engaging in risky sex behavior could be the wish to boost one’s self-esteem (Layh et al., 2020). These affect or self-esteem regulatory sex motives might be a factor linking childhood maltreatment with sexual victimization in the context of online dating, too, and are thus worth investigating.

Interestingly, in-person sexual victimization might be preceded by cyber victimization indicated by a previous study, which detected a strong association between in-person and cyber sexual victimization in female adolescents (Zetterström Dahlqvist & Gillander Gådin, 2018). Thus, it will be informative to study whether cyber victims decide to meet their matches in person despite their awareness of the risk and whether this is moderated by affect/self-esteem regulatory sex motives. Victims of cyber victimization who engage in risky situations due to a stronger urgency to avoid negative feelings or feel better about themselves via sex might have an increased risk of in-person sexual victimization compared to victims with moderate or low levels of affect/self-esteem regulatory sex motives.

Although risky sex behavior such as casual sex is common in online dating (Bryant & Sheldon, 2017; Timmermans & Courtois, 2018), there is evidence that the users are aware of the risks of online dating, including the risk of sexual victimization (Couch et al., 2012). Therefore, people might apply protective strategies like sharing the meeting point of the first date with family or friends as an attempt to stay safe. Nevertheless, people with high affect/self-esteem regulatory sex motives might prioritize these motives in their decision-making and use fewer protective strategies. Knowledge about the association between the frequency of employing such protective strategies and sex motives is one of the gaps in the literature.

The current study aims to further our knowledge about the predictors of adult sexual victimization and revictimization among online dating users by testing several hypotheses: A. Based on the study by Zetterström and colleagues (2018), we hypothesize that cyber sexual victimization severity is positively related to in-person sexual victimization severity. B. Based on previous studies, we assume that childhood maltreatment severity is positively related to both cyber and in-person sexual victimization severity in adulthood. C. We assume that affect/self-esteem regulatory sex motives will mediate the relationship between child maltreatment severity and in-person sexual victimization severity during adulthood, and these motives moderate the relationship between cyber sexual victimization severity and in-person sexual victimization severity. D. We hypothesize that affect/self-esteem regulatory sex motives are negatively associated with the use of protective dating strategies.

## Method

### Participants

Heterosexual women (*N* = 523) aged between 18 and 35 who had used mobile dating applications in the year before the assessment and met at least one of their matches in person were recruited by Qualtrics Company (*N* = 373) or a research platform at the University of Groningen (*N* = 150), the Netherlands. The former recruited participants from the general population and the latter university students. To assure that a proper number of people with a history of childhood maltreatment was included in the sample, only people indicating a positive history of childhood maltreatment via a dichotomous item (“Were you emotionally abused or neglected as a child (before the age of 15) or did you suffer any form of sexual or physical abuse during your childhood?”) were included in the general population sample. In total, 110 responses were excluded (see more information in the data analysis section). The final sample included 413 participants (*n* = 276 general population and *n* = 137 university students), of whom 83.8% (*n* = 346) were Dutch, 8% (*n* = 33) were German, and the remaining (*n* = 34, 8.2%) were from various countries. The mean age of the participants was 23.68 (*SD* = 3.62) years. The participants consented to the study before responding to the survey and received research credits or a monetary reward depending on the platform via which they participated. The survey took approximately 20 minutes. The study was approved by the Ethics Committee at the University of Groningen and preregistered at aspredicted.org under nr. 56,818.

### Measures

#### Demographic and Mobile Dating Applications Information

The participants reported their age, nationality, relationship status, main motivation for using mobile dating applications, the number of matches met in person, duration of application use, how often they engaged in sexual activities with a new partner on the first date, and the last time they met a match in person (see Table 1).

**Table 1.** Demographic and Mobile Dating Applications Information.

	*N* (%)		Not Reported *N* (%)
**Relationship status**			29 (7)
Single	335 (81.1)		
In a relationship - partner knows one uses the apps	37 (9)		
In a relationship - partner does not know one uses the apps	12 (2.9)		
**Motives for using the apps**			9 (2.2)
Serious relationship	207 (50.1)		
Casual sex	54 (13.1)		
Meeting new people and making friends	143 (34.6)		
	**Mean (*SD*)**	**Range (mode)**	
**Age**	23.68 (3.62)	18–35 (25)	2
**Duration on the apps (in month)**	13.19 (14.38)	1–200 (12)	18
**No of matches met in person**	7.74 (8.51)	1–70 (3)	13
**Last time dated a match in person (in days)**	63.88 (104.57)	0–700 (10)	42
**Sex on the first date**	.91 (2.03)	0–20 (0)	13

#### Childhood Maltreatment

To measure childhood maltreatment, Childhood Trauma Questionnaire-Short Form (CTQ-SF; Bernstein et al., 2003) with 28 items was administered. This scale has five subscales of emotional abuse, emotional neglect, physical abuse, physical neglect, and sexual abuse consisting of five items each. The participants were instructed to indicate how frequently they experienced these maltreatments before the age of 15 on a 5-point Likert scale (1 = never true to 5 = very often true). If they were not willing to report them, they could choose “I do not wish to answer this question” option that was added to the scale. The CTQ-SF has shown proper psychometric features in different countries and populations (Bernstein et al., 2003; Gerdner & Allgulander, 2009; Thombs et al., 2009). The Cronbach’s alpha of the scale in this sample was .94. Sum scores for each subscale were computed by summing up the values for the corresponding items and total scores for the whole scale were computed by summing up the 25 subscale items, leaving out three validity items. The cut-offs proposed by Walker et al. (1999), sexual abuse ≥8, physical abuse ≥8, physical neglect ≥8, emotional neglect ≥15, and emotional abuse ≥10, were used to understand the number of individuals with childhood abuse severity above the cut-off for each subscale.

#### Sexual Victimization in Adulthood

We created 10 items to measure sexual victimization in the context of online dating, two items for cyber sexual victimization and eight items for in-person victimization. The participants were instructed to indicate the number of cases in which they were victimized by their matches using a visual analog scale (0 = 0% or never, 100 = 100% or in all cases). Examples of the items are “My match sent me unwanted sexual texts although I had clearly told him I did not like that” and “My match kissed me although I had clearly told him I did not like that.” Sexual victimization ranged from non-consensual kissing to rape (see Table 2). The Cronbach’s alpha of the scale was .95.

**Table 2.** Frequency of Cyber and In-person Adult Sexual Victimization.

	Mean	*SD*	*N* (%) of Victims	Not Reported
My match sent me unwanted sexual texts although I had clearly told him I did not like that	27.52	30.30	195 (47.2)	120
My match sent me nudes although I had clearly told him I did not like that	24.06	29.57	184 (44.6)	121
My match kissed me although I had clearly told him I did not like that	17.92	27.37	166 (40.2)	122
My match touched a part of my body although I had clearly told him I did not like that	18.58	26.34	160 (38.7)	122
My match encouraged me to use drugs and then had sex with me without my consent/permission	12.79	24.21	126 (30.5)	123
My match encouraged me to drink more than I wanted and then had sex with me without my consent/permission	14.28	25.04	139 (33.7)	122
I had sex with my match because my match threatened me that he would leave me if I did not have sex with him	11.72	23.07	131 (31.7)	125
My match made me have sex with him in a way that I did not want	17.55	28.61	158 (38.3)	124
My match made me have sex with him without protection	18.47	29.02	146 (35.4)	125
My match treated me in a way that made me feel really uncomfortable and went beyond what I had agreed to do	18.99	28.26	139 (33.7)	123

To determine the number of people sexually victimized by their matches, cut-offs were created: Indication of at least one percent on at least one of the corresponding items was considered as cyber-sexual or in-person victimization, respectively. To compute cyber and in-person sexual victimization severity in adulthood, the percentages on the corresponding items were summed up (Table 3). In addition, we divided the item of this scale with the highest percentage by the number of matches met in-person to determine the minimal number of separate incidents. The mean of separate incidents was 31.80 (*SD* = 34.55) with a range between 0 and 100.

**Table 3.** Mean, Standard Deviations, Range, and the Number of Cases for the Study Variables.

	Mean	SD	Range	*N*
**Childhood maltreatment**	46.78	19.38	25–105	350
Emotional abuse	11.12	5.38	5–25	407
Physical abuse	8.64	4.53	5–23	401
Sexual abuse	8.20	5.09	5–25	395
Emotional neglect	10.61	4.72	5–25	384
Physical neglect	8.66	4.01	5–22	396
Sexual victimization severity during online dating				
Cyber victimization	51.64	56.92	0–200	292
In-person victimization	130.56	180.47	0–713	288
**Approach-avoidance sex motives**	461.92	251.01	3–1100	149
**Protective dating strategies**	15.91	3.49	7–20	407
Motives for online dating				
Entertainment	399	35.23	0–70	17.36
Social approval	398	27.85	0–60	15.07
Relationship seeking	401	26.90	0–50	12.81
Flirting	399	22.70	0–53	13.39
Socialization	400	18.96	0–40	9.97
Sexual experience	391	17.52	0–60	14.82
Peer pressure	399	9.71	0–30	7.92
Get over Ex	395	9.09	0–30	8.87

#### Motives for Casual Sex in Online Dating Scale

We used five items from the Motivations for Sexual Intercourse Scale (Cooper et al., 1998) to assess affect regulation motive and we added six custom-made items measuring self-esteem regulation motive. This measure was administered for the participants who indicated having casual sex with their matches (*n* = 158). The participants indicated the percentage of the cases in which they had casual sex with their matches with those motives on a visual analog scale (0 = 0% or never, 100 = 100% or in all cases). The examples of the items were “I have casual sex with matches because I would like to be adventurous” for self-esteem regulation motive and “I have casual sex to cope with upset feelings” for affect regulation motive. The Cronbach’s alpha of the Motives for Casual Sex in Online Dating Scale (under submission) in the sample was 0.91. To compute the total scores, we summed the values on the corresponding items (Table 3).

#### Protective Dating Strategies

Protective dating strategies were assessed by two sets of three items each which had some content overlap. Participants rated as the percentage of cases in which they used those strategies when meeting their matches in person on a visual analog scale (0 = 0% or never, 100 = 100% or in all cases). Of these, one item was adapted from the Dating Behavior Survey (Breitenbecher, 2008), three from the Dating Self-Protection Against Rape Scale (Moore & Waterman, 1999) and were modified for online dating, while the remaining two items were custom made (see Supplementary Table). An example is “I shared my match’s phone number with a friend or family before I met my match in person.” Since it was not clear if the reported strategies were applied for the same or different dates, we recoded the values to 1–10. Items one to three and items four to six had overlaps in content. Thus, we selected the item with the highest values per item set (items 1–3 and 4–6). Next, the sum score of these two items was computed, which is presented in Table 3.

#### Motives for Dating

Motives for using mobile dating applications were measured by eight subscales of the Tinder Motives Scale (Timmermans & De Caluwé, 2017), that is, Social Approval, Relationship Seeking, Sexual Experience, Flirting/Social Skills, Ex-Partner, Peer Pressure, Socializing, Pass Time/Entertainment consisting of 40 items. This scale has shown good psychometric properties (Timmermans & De Caluwé, 2017). The participants reported their motivations on a visual analog scale (0 = 0% or never, 100 = 100% or in all cases) on statements such as, “I use online dating applications to get an ego boost.” Sum scores were computed for each subscale (Table 3). The Cronbach’s alpha of the subscales in our sample was ranging from .85 for Socialization to .95 for Ex-Partner.

### Data Analysis

#### Data Cleaning

There were 79 participants who terminated their participation during the multi-step consent procedure. Since these participants did not provide any information, they were removed from further analyses. In total, 19 participants provided duplicate responses, of which only the first entry was always retained. In addition, we removed 12 participants who showed response patterns such as the same response to all items of a scale or consecutive numbers repetitively such as numbers from 1 to 5.

#### Imputation of Missing Values Estimation

No missing values were imputed, except for one item for adulthood sexual victimization of one participant that was imputed by the mean of nearby values, due to reasons explained below. Hence, participants were removed pairwise from the analyses depending on their missing values on each measure. Imputation of missing values was precluded by either missing not being random (Childhood Trauma Questionnaire and the MOCS), by more than 10% of the values missing per scale (Sexual Victimization in Adulthood Scale), or by inter-item correlations not being sufficiently large, that is, less than .20, indicating that the items are not good predictors of each other (Protective Dating Strategy Scale).

**Assumption check and statistical tests:** The assumptions of linearity and independence of residuals for linear regression were met. The assumptions of homoscedasticity and normality of residuals for regression were not met for all variables. However, since the violations of these assumptions do not have severe consequences in large samples (Ernst & Albers, 2017), regression analyses were carried out. The Process Macro v3.5 (Hayes, 2012) was employed for mediation and moderation analyses with 10,000 bootstrapping samples. Data cleaning and analyses were conducted in SPSS 25.

## Results

### Descriptive Results

The majority of participants were single (*N* = 335, 81.1%), approx. half of the participants (*N* = 207) were looking for a serious relationship in online dating and approx. 30% for meeting new people or finding new friends. At the time of the study, they had used mobile dating applications on average for 13.19 months (*SD =* 14.38*).* The number of matches met in person ranged from 1 to 70 with a mode of 3. The number of matches with whom participants engaged in sexual interactions on the first date ranged from 0 to 20 with the mode of 0. The two most common motives for using dating applications were passing time and receiving social approval from matches and the two least common motives were getting over one’s ex-partner and peer pressure.

The percentage of people in the sample reporting emotional neglect was 20.1% (*n* = 83), emotional abuse 43.8% (*n* = 181), sexual abuse 32.2% (*n* = 133), physical abuse 37.0% (*n* = 153), and physical neglect 45.8% (*n* = 189). In total, 56.3% of the participants (*n* = 232) reported at least one type of childhood maltreatment.

In the whole sample, 49.2% (*n* = 203) reported at least one type of cyber sexual victimization and 52.1% (*n* = 215) at least one type of in-person sexual victimization in the context of online dating (see Table 2). Furthermore, 32% (*n* = 132) reported both childhood maltreatment and cyber-sexual victimization and 35.4% (*n* = 146) reported both childhood maltreatment and in-person sexual victimization. Any form of revictimization was reported by 36.8% (*n* = 152). Sexual revictimization, defined as sexual abuse in childhood and in-person sexual victimization in adulthood, was reported by 23% of the sample (*n* = 95).

### Hypothesis Testing

As hypothesized, cyber sexual victimization severity was positively associated with in-person sexual victimization severity (*β* = 2.28, *t* (286) = 17.35, *p* < .001) with an effect size of R^2^ = .50, *F* (1, 286) = 300.94, *p* < .001.

Childhood maltreatment severity was also positively related to cyber sexual victimization severity (*β* = 1.30, *t* (256) = 8.66, *p* < .001) with an effect size of R^2^ = .23, *F* (1, 256) = 75, *p* < .001.

In line with previous studies, childhood maltreatment severity was positively related to in-person sexual victimization severity (*β* = 5.24, *t* (252) = 12.67, *p* < .001) with an effect size of R^2^ = .39, *F* (1, 252) = 160.57, *p* < .001.

**Figure 1.**
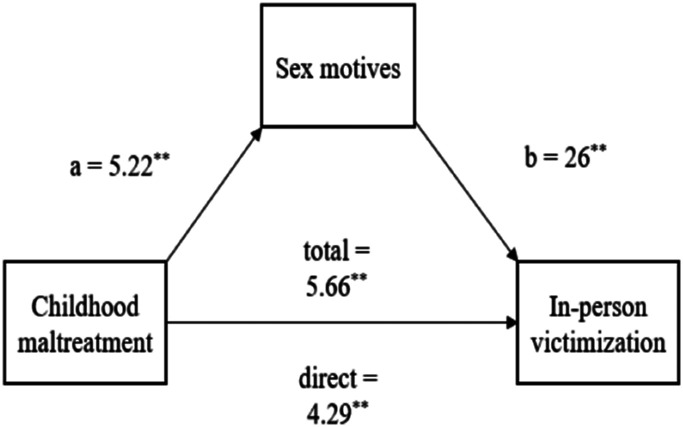
Mediating Effect of Approch-Avoidance Sex Motives on in Person Sexual Victimization.

Affect/self-esteem regulatory sex motives mediated the relationship between childhood maltreatment severity and in-person sexual victimization severity (*β* = 1.37, 95% *CI* [.62, 2.26]).Figure 1 presents the paths of the model.

**Figure 2.**
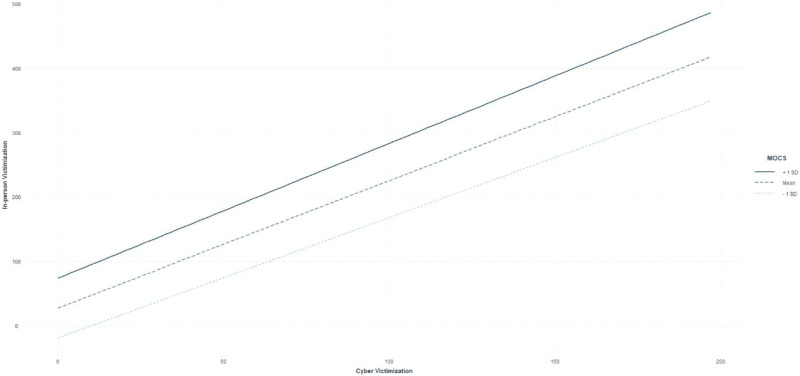
Estimated Coefficients for Adult Sexual Victimization on Cyber Victimization by Level of MOCS.

Affect/self-esteem regulatory sex motives moderated the association between cyber and in-person sexual victimization severity indicated by a significant interaction (*β* = .002, *t* (140) = 3.58, *p* < .001). The association between cyber and in-person sexual victimization was significant at low (*β* = 1.02, *t* (140) = 4.08, *p* < .001), moderate (*β* = 1.66, *t* (140) = 9.20, *p* < .001) and high (*β* = 2.29, *t* (140) = 9.01, *p* < .001) levels of affect/self-regulatory sex motives. However, as these motives increase, the effect of cyber victimization on in-person sexual victimization becomes stronger as presented in Figure 2.

The affect/self-esteem regulatory sex motives were not associated with protective dating strategies (*β* = −.002, *t* (147) = −1.69, *F* (1, 147) = 2.85, *p* = .09).

## Discussion

The aim of the current investigation was to understand whether childhood maltreatment severity is related to sexual victimization in adulthood among mobile dating application users—and whether motives for casual sex mediate this association.

The findings indicate that childhood maltreatment is a risk factor for revictimization in online dating, too. Using sex to regulate negative emotions and self-esteem links childhood maltreatment to sexual victimization in adulthood. Cyber victimization and affect/self-esteem regulatory sex motives show an interaction effect on in-person sexual victimization with a stronger effect of cyber victimization on in-person sexual victimization as the levels of those sex motives increase. In addition, affect/self-esteem regulatory sex motives were not associated with employing fewer protective strategies.

### Associations Between Childhood and Adulthood Victimization

Greater severity of childhood maltreatment was related to higher severity of both cyber and in-person sexual victimization in the present study. These findings are in line with previous studies showing a relationship between childhood maltreatment and victimization in adulthood (Draucker, 1997; Gidycz et al., 1993; Hocking et al., 2016). Thus, our results indicate that childhood maltreatment also increases the likelihood of sexual victimization in online dating similar to other contexts.

In addition, the association between victimization in childhood and adulthood was mediated by affect/self-esteem regulatory sex motives in the current study. Higher childhood maltreatment severity was related to higher affect/self-esteem regulatory sex motives, which in turn were related to higher severity of in-person sexual victimization. This replicates the finding by Miron and Orcutt (2014) that a need to regulate strong negative affect can be a motive to engage in casual sex and can thus act as a risk factor for revictimization.

These findings are also in line with the theoretical conceptualizations by Finkelhor (1988) and Orcutt et al. (2005) regarding sexual revictimization, which assume that the survivors of childhood maltreatment might engage in risky sex behaviors to meet non-sexual goals such as emotion regulation or interpersonal goals like receiving attention from others, which in turn might increase the risk of revictimization. Therefore, it seems important to further investigate the association between motives for active engagement in risky situations and revictimization since few studies have investigated this so far.

### The Role of Non-Sexual Motives

The mechanism linking affect or self-esteem regulatory motives to sexual revictimization has not been extensively studied yet. Our findings could, for example, indicate that people with childhood maltreatment history who use sex to regulate their emotions or to boost their self-esteem might be less selective in their partner selection, might consciously accept certain risks, or might not become aware of indicators of risk. Miron and Orcutt (2014) found that the intervening factor between using sex to reduce negative affect and sexual victimization in adulthood was sex with strangers. In the current study, the main motivation for online dating was not casual sex and the majority of the sample did not report sex on the first date although online dating is commonly used for casual sex. Therefore, the mechanism in this sample might be through other risky sex behavior such as sex under the influence of alcohol/substance or higher number of sexual partners. These links need to be studied in future studies. In addition, since this study assessed exclusively intrapersonal motives for casual sex, it might be informative to examine whether social motives for casual sex proposed by Cooper et al. (1998), using sex to avoid social rejection or to feel connection with someone, mediate the association between childhood maltreatment and revictimization. In line with this assumption, the participants indicated using the applications for social motives, mainly for social approval and relationship seeking. Thus, affiliation might be a driving motivation for using online dating, which in turn might be related to affect and self-esteem regulation needs.

### The Link Between Cyber and In-Person Victimization

The significant relationship between cyber and in-person sexual victimization found in this study indicates that women sexually victimized in the virtual environment are at risk of further sexual victimization in person. This evidence is in line with a prior study (Zetterström Dahlqvist & Gillander Gådin, 2018) reporting an association between in-person and cyber sexual victimization and extend the previous finding to an adult population. The association between these two forms of victimization could either be due to victim selection on the side of the perpetrator, shared underlying mechanisms on the side of the victim, or an interaction of both. For instance, non-assertiveness or ambiguous communication in response to cyber victimization might signal to the perpetrator that a further transgression will meet little resistance and thus might be the shared factors linking victimization in cyber and in-person contexts.

Our findings showed that the associated between cyber and in-person sexual victimization is moderated by the affect/self-esteem regulatory sex motives. Cyber victimization was positively associated with in-person sexual victimization at different levels of affect/self-esteem regulatory sex motives. However, the association was stronger as those motives increased. It can be concluded that cyber victims are at the risk of in-person sexual victimization even when they use sex as an emotion/self-esteem regulatory strategy at the minimum level. Furthermore, higher levels of such motives might put cyber victims even at greater risk of in-person sexual victimization compared to lower levels. Therefore, cyber victims might decide to meet potential perpetrators in person due to urgent need to regulate negative emotions or boost self-esteem. Further research on victims being perpetrated by the same person in cyber and in-person contexts can test this assumption in future research.

### Safety Measures

Higher affect regulatory sex motives were not significantly related to less effort to stay safe in online dating although the direction of association was negative. Since this is the first study conducted on this association, further research is needed to understand if those sex motives influence the extent to which people try to decrease the risk of sexual victimization in online dating. Future qualitative studies assessing protective strategies people use in online dating can result in designing a valid measure examining those strategies and, then their relationships with affect/self-esteem regulatory sex motives.

**Strengths.** This is the first study on the factors related to sexual victimization in online dating and it included both community and university student samples. Unlike most studies in the field of revictimization that had been conducted in the USA, the present study was conducted in Europe. Another asset of the study was measuring the effect of self-esteem regulation as a sex motive while previous studies only measured affect regulatory motive of sex.

In our recruitment, we tried to artificially increase the proportion of participants with a history of childhood trauma in order to be able to establish the associations between trauma experiences and sex motives well. Our recruitment strategy was successful in this regard as indicated by a higher prevalence of childhood maltreatment in our sample (56.3%) than in the general population (35% in the Netherlands as reported by the European Union Agency for Fundamental Rights (FRA; 2014) for the combined prevalence of childhood physical, emotional, and sexual abuse).

The overall revictimization rate in this study was 36.8%, the rate of sexual revictimization following sexual childhood abuse specifically was 23%. These rates are close to the 30% rate of sexual revictimization in a study by West et al. (2000), which examined a sample with documented history of child sexual abuse. However, the rate of sexual revictimization is lower in the present study compared to another study in the Netherlands with 50% rate of sexual revictimization in women (de Haas et al., 2012). The age range in their sample was larger (between 15 and 70 years old), which might have resulted in the higher rate.

#### Limitations

The sample of the study is limited to heterosexual women in early adulthood and the results are not generalizable to homosexual individuals, men or younger or older populations. In addition, the cross-sectional design of the study limits the interpretations about the causal relationship between the variables. For instance, it can be discussed that the affect/self-esteem regulatory sex motives are not only the precursor but also the results of sexual victimization in adulthood. The fact that we did not find a significant association between these motives and the use of protective strategies could be due to the fact that the latter were not assessed by a validated and comprehensive measure. Future studies should aim at developing such a measure and also assess to which extent users of dating apps have realistic risk estimates for the context of online dating.

We were not able to apply the same inclusion criteria in both subsamples, which could have led to a systematic effect on the composition of the group which scored above the cut-off for childhood trauma. However, we did not detect any significant differences regarding the duration of using the app, number of dates met in person, relationship status, or main motives for online dating, but we cannot rule out that there might be differences in sample composition on factors which we did not assess in the current study. A higher percentage of subjects with a history of childhood maltreatment reported sex on the first date than subjects below the cut-off for childhood maltreatment, but we cannot rule out that this was simply due to their slightly higher age (see Supplementary Table). However, as our main results are not based on a comparison of these subgroups, the difference in recruitment strategy should not have influenced our data very much. More importantly, we cannot ascertain that our sample is representative of the population as we do not have any data on subjects who were invited to participate (or saw the study description on the recruitment website) and declined due to the content of the study, which could have led to a recruitment bias. It is both conceivable that subjects with victimization experiences were particularly interested in the study as well as that they avoided exposure to this topic at a higher rate. Thus, a replication in a representative sample of app-users would be helpful.

#### General conclusion

Heterosexual young women with a history of childhood maltreatment are at higher risk of sexual victimization in adulthood in the context of online dating. Using sex to reduce negative affect or to boost self-esteem is one of the factors linking childhood maltreatment to higher risk of revictimization. These sex motives play a moderating role in the relationship between cyber and in-person sexual victimization. Since this study was the first study exploring the factors related to sexual victimization in online dating, further investigation is needed. Future studies should aim at replicating these associations prospectively. If future studies show similar results, interventions addressing motives underlying online dating use, particularly for casual sex, might be able to decrease the risk of sexual victimization especially in individuals with a history of childhood maltreatment.

## Supplemental Material

sj-pdf-1-jiv-10.1177_08862605211073715 – Supplemental Material for Predictors of Revictimization in Online Dating Click here for additional data file. Supplemental Material, sj-pdf-1-jiv-10.1177_08862605211073715 for Predictors of Revictimization in Online Dating by Fatemeh Fereidooni, Judith Daniels, and Miriam Lommen in Journal of Interpersonal Violence
