# Supplementary material for: Predictors of Revictimization in Online Dating
Source: J Interpers Violence. 2022 Feb 28;37(23-24):NP23057–74. doi: 10.1177/08862605211073715 (PMC9679550; doi:10.1177/08862605211073715)
Supplement: sj-pdf-1-jiv-10.1177_08862605211073715 – Supplemental Material for Predictors of Revictimization in Online Dating [file sj-pdf-1-jiv-10.1177_08862605211073715.pdf]

Table S1. Protective Dating Strategies Items

1.	I let a friend or family know where and when I would meet my match.
2.	I shared my match's photo with a friend or family before I dated my match in person to make sure they knew who I would meet.
3.	I shared my match's phone number with a friend or family before I met my match in person.
4.	I met my match in public (for example, a café or a restaurant) for the first date even when I did want to have casual sex with him
5.	On our first date, I had my match pick me up at home.
6.	On the first date with my match, I chose our activities or meeting point.

Table S2. Supplementary Analyses on the Differences Between People Above and Below Cut-offs for Childhood Maltreatment

	**N (%) Below cut-off**	**N (%) Above cut-off**	**Difference**
**Relationship status**			
Single	99 (91.7)	184 (84.4)	(X^2 (2, *n* = 326) = 4.92, p = .09)
In a relationship[1]	5 (4.6)	27 (12.4)	
In a relationship[2]	7 (3.2)	7 (3.2)	
**Main motive for online dating**			
Serious relationship	49 (42)	119 (52.2)	(X^2 (2, *n* = 342) = 2.64, p .27)
Casual sex	18 (15.8)	32 (14)	
Meet people or make friends	47 (42.2)	77 (33.8)	
**Sex on the first date**			
No	88 (75.9)	132 (58.9)	(X^2 (1, *n* = 340) = 9.60, p = .002)
Yes	28 (24.1)	92 (41.1)	
	**M (*SD*)**	**M (*SD*)**	
**Duration of using apps**	13.33 (12.44)	13.13 (16.16)	(*t* (333) = .11, *p* = .90)
**Met dates in-person**	7.44 (8.14)	7.39 (7.91)	(*t* (338) = .05, *p* = .96)
**Age**	22.50 (2.93)	24.03 (3.86)	(*t* (294.46) = -4.12, *p* < .001)

1. The partner knows that the participant is dating other people
2. The partner does not know that the participant is dating other people
